# Low-Molecular-Weight Chitosan Attenuates Lipopolysaccharide-Induced Inflammation in IPEC-J2 Cells by Inhibiting the Nuclear Factor-κB Signalling Pathway

**DOI:** 10.3390/molecules26030569

**Published:** 2021-01-22

**Authors:** Jiao Zhang, Jin Wan, Daiwen Chen, Bing Yu, Jun He

**Affiliations:** Institute of Animal Nutrition, Sichuan Agricultural University, Chengdu 611130, China; zjpm25@163.com (J.Z.); wanjin91@163.com (J.W.); dwchen@sicau.edu.cn (D.C.); ybingtian@163.com (B.Y.)

**Keywords:** low-molecular-weight chitosan, lipopolysaccharide, inflammatory injury, cell apoptosis, intestinal epithelial cells

## Abstract

Low-molecular-weight chitosan (LMWC), a product of chitosan deacetylation, possesses anti-inflammatory effects. In the present study, a porcine small intestinal epithelial cell line, IPEC-J2, was used to assess the protective effects of LMWC on lipopolysaccharide (LPS)-induced intestinal epithelial cell injury. IPEC-J2 cells were pretreated with or without LMWC (400 μg/mL) in the presence or absence of LPS (5 μg/mL) for 6 h. LMWC pretreatment increased (*p* < 0.05) the occludin abundance and decreased (*p* < 0.05) the tumour necrosis factor-α (TNF-α) production, apoptosis rate and cleaved cysteinyl aspartate-specific protease-3 (caspase-3) and -8 contents in LPS-treated IPEC-J2 cells. Moreover, LMWC pretreatment downregulated (*p* < 0.05) the expression levels of TNF receptor 1 (*TNFR1*) and TNFR-associated death domain and decreased (*p* < 0.05) the nuclear and cytoplasmic abundance of nuclear factor-κB (NF-κB) p65 in LPS-stimulated IPEC-J2 cells. These results suggest that LMWC exerts a mitigation effect on LPS-induced intestinal epithelial cell damage by suppressing TNFR1-mediated apoptosis and decreasing the production of proinflammatory cytokines via the inhibition of NF-κB signalling pathway.

## 1. Introduction

The intestinal epithelial barrier is mainly composed of epithelial cell membranes and tight junctions between epithelial cells; it can prevent foreign antigens, pathogens and toxins from entering the circulatory system [1,2]. Hence, an intact intestinal barrier plays an important role in maintaining intestinal homeostasis [3]. However, many harmful substances, such as lipopolysaccharides (LPSs), can cause intestinal epithelial cells to secrete large amounts of proinflammatory cytokines, thereby impairing the intestinal barrier integrity [4,5]. In addition, the secreted proinflammatory cytokines can expedite the apoptosis of intestinal epithelial cells and further damage the intestinal barrier integrity [6,7]. Therefore, dietary treatments targeted at reducing the intestinal proinflammatory cytokine production and epithelial cell apoptosis could contribute to improving the intestinal barrier integrity.

Chitosan, a deacetylated product of chitin, is the major structural component in the exoskeletons of insects, crustaceans and arachnids [8]. It is a non-toxic, non-immunogenic and biodegradable biomedical material that is widely used in humans and animals [9,10,11,12]. However, chitosan extracted from chitin has a high molecular weight and poor solubility, which greatly limit its applications. To address these undesirable properties, more active forms, such as chitosan oligosaccharide and low-molecular-weight chitosan (LMWC), have been generated [13]. LMWC possesses various biological properties, including antimicrobial [14], antiapoptotic [15], antioxidant [16] and anti-inflammatory [17] properties. Recently, LMWC has been shown to be beneficial in enhancing the intestinal barrier function in weaned pigs [18]. However, little is known about whether LMWC has a protective effect on LPS-induced intestinal barrier damage. Hence, the present study aimed to assess the protective effects of LMWC on LPS-induced intestinal epithelial cell injury and its potential mechanisms using a porcine small intestinal epithelial cell line, IPEC-J2.

## 2. Results

### 2.1. LPS Stimulation Dose and Time

**Figure 1:**
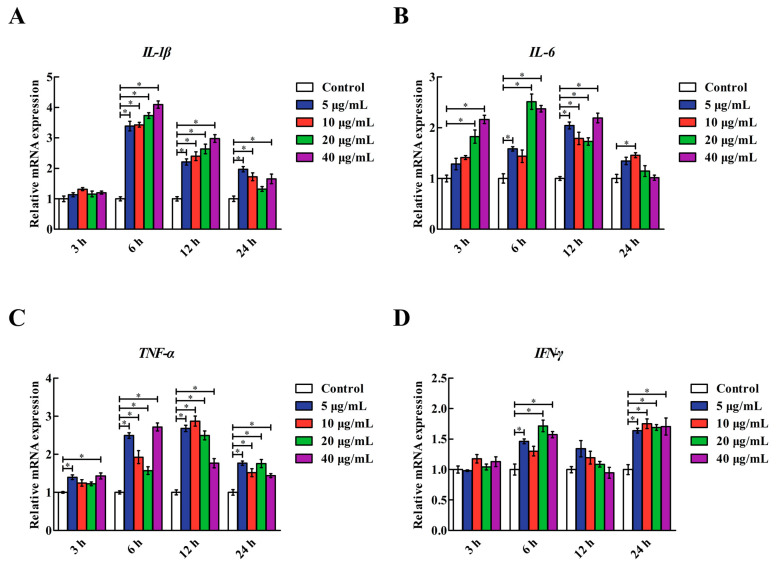
Effects of various LPS concentrations and reaction times on the *IL-1β*, *IL-6*, *TNF-α* and *IFN-γ* mRNA levels in IPEC-J2 cells. (**A**) *IL-1β*: interleukin-1β. (**B**) *IL-6*: interleukin-6. (**C**) *TNF-α*: tumour necrosis factor-α. (**D**) *IFN-γ:* interferon-γ. Cells were treated with various concentrations of LPS (0, 5, 10, 20 and 40 μg/mL) for 3, 6, 12 and 24 h. Data are presented as the mean ± standard error (*n* = 4). * Means significant difference compared with the control group (*p* < 0.05).

As shown in Figure 1, LPS stimulation upregulated (*p* < 0.05) the interleukin-1β (*IL-1β*), *IL-6*, tumour necrosis factor-α (*TNF-α*) and interferon-γ (*IFN-γ*) mRNA levels in the IPEC-J2 cells in a dose- and time-dependent manner. Of the doses and times tested, treatment with 5, 20 and 40 μg/mL LPS for 6 h resulted in the upregulation (*p* < 0.05) of *IL-1β*, *IL-6*, *TNF-α* and *IFN-γ* mRNA levels in the IPEC-J2 cells; the effects of those three treatments were similar. Therefore, IPEC-J2 cells treated with 5 μg/mL LPS for 6 h were used in subsequent experiments for the induction of inflammatory damage in intestinal epithelial cells.

### 2.2. LMWC Dose

**Figure 2:**
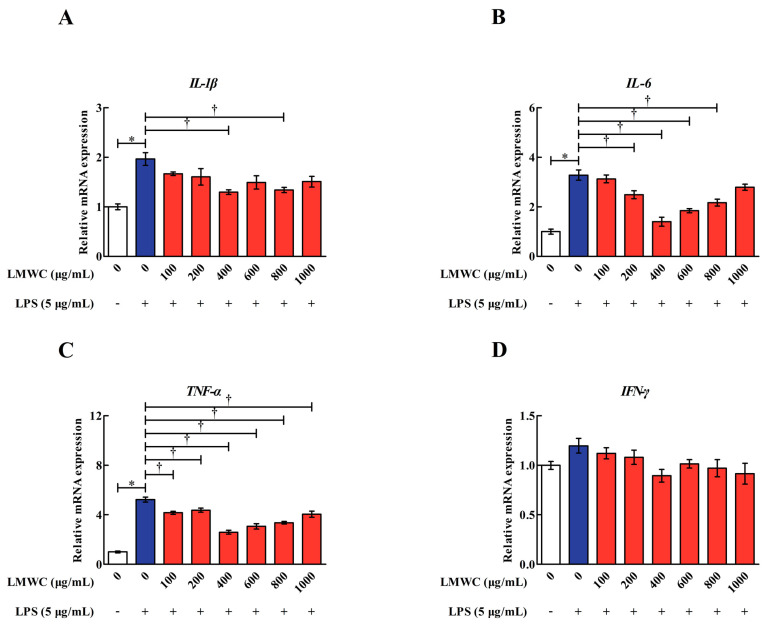
Relative *IL-1β*, *IL-6*, *TNF-α* and *IFN-γ* mRNA levels in LPS-treated IPEC-J2 cells after pretreatment with different concentrations of LMWC. (**A**) *IL-1β*: interleukin-1β. (**B**) *IL-6*: interleukin-6. (**C**) *TNF-α*: tumour necrosis factor-α. (**D**) *IFN-γ*: interferon-γ. Cells were pretreated with various concentrations of LMWC (0, 100, 200, 400, 600, 800 and 1000 μg/mL) for 2 h, followed by cotreatment with LPS (5 μg/mL) for another 6 h. Data are presented as the mean ± standard error (*n* = 4). * Means significant difference compared with the control group (*p* < 0.05). ^†^ Means significant difference compared with the LPS group (*p* < 0.05).

For choosing an appropriate LMWC dose against LPS-induced inflammatory damage in IPEC-J2 cells, we evaluated the mRNA levels of *IL-1β*, *IL-6*, *TNF-α* and *IFN-γ* in the LPS-treated IPEC-J2 cells. On pretreating the IPEC-J2 cells with 100–1000 μg/mL LMWC for 2 h, we found that 400 μg/mL LMWC was the most effective in reducing (*p* < 0.05) the mRNA levels of *IL-1β*, *IL-6* and *TNF-α* in the LPS-treated IPEC-J2 cells (Figure 2). Hence, the optimal LMWC concentration for the subsequent experiment was confirmed to be 400 μg/mL.

### 2.3. Cytokine Concentration

**Figure 3:**
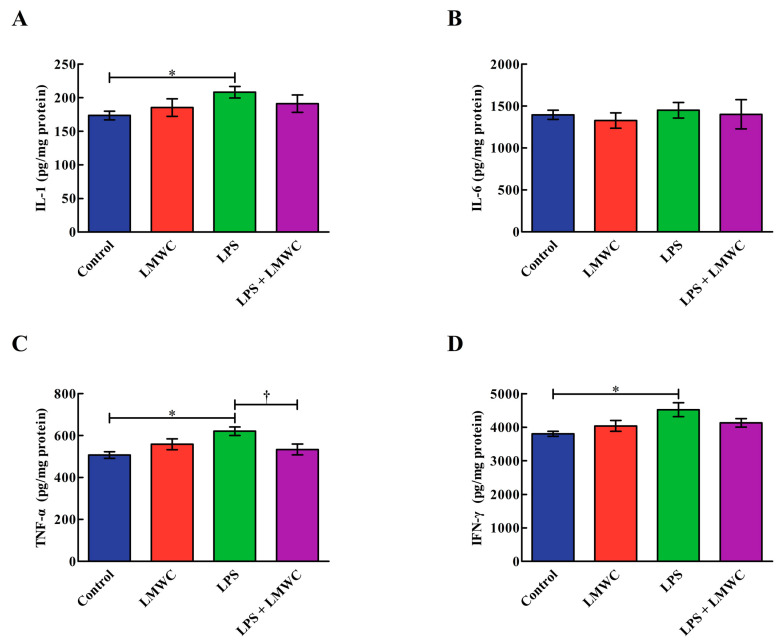
Effects of LMWC on the proinflammatory cytokine concentrations in LPS-treated IPEC-J2 cells. (**A**) IL-1: interleukin-1. (**B**) IL-6: interleukin-6. (**C**) TNF-α: tumour necrosis factor-α. (**D**) IFN-γ: interferon-γ. Cells were pretreated with LMWC (400 μg/mL) for 2 h, followed by cotreatment with LPS (5 μg/mL) for another 6 h. Data are presented as the mean ± standard error (*n* = 4). * Means significant difference compared with the control group (*p* < 0.05). ^†^ Means significant difference compared with the LPS group (*p* < 0.05).

As shown in Figure 3, the addition of LPS increased (*p* < 0.05) the IL-1, TNF-α and IFN-γ concentrations in the IPEC-J2 cells, while LMWC pretreatment decreased (*p* < 0.05) the TNF-α concentration in the LPS-treated IPEC-J2 cells. Moreover, no differences (*p* > 0.05) were noted in the IL-6 concentration among the four groups.

### 2.4. Occludin Abundance

**Figure 4:**
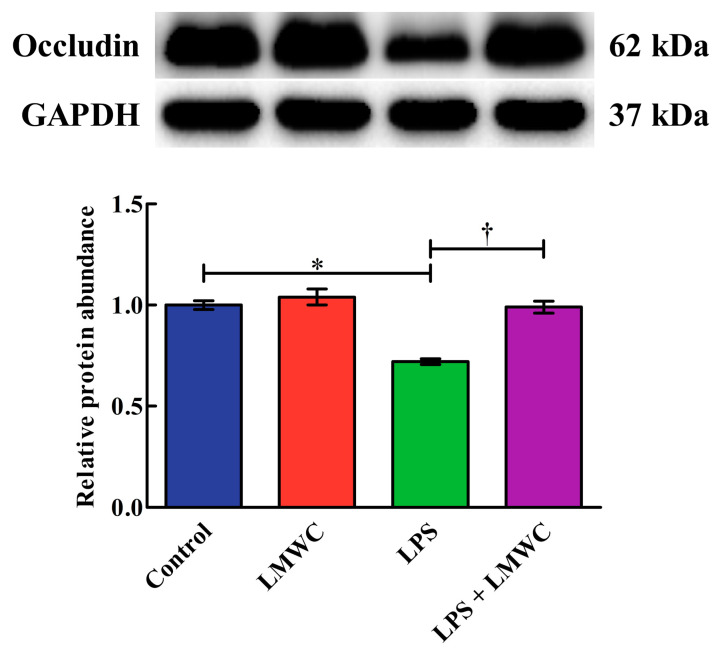
Effects of LMWC on the occludin abundance in LPS-treated IPEC-J2 cells. Cells were pretreated with LMWC (400 μg/mL) for 2 h, followed by cotreatment with LPS (5 μg/mL) for another 6 h. Data are presented as the mean ± standard error (*n* = 4). * Means significant difference compared with control group (*p* < 0.05). ^†^ Means significant difference compared with the LPS group (*p* < 0.05).

The effects of LMWC on occludin abundance in the LPS-treated IPEC-J2 cells are shown in Figure 4. The cells treated with LPS showed reduced (*p* < 0.05) occludin abundance. Importantly, LMWC pretreatment increased (*p* < 0.05) the occludin abundance in the LPS-treated IPEC-J2 cells.

### 2.5. Cell Apoptosis

**Figure 5:**
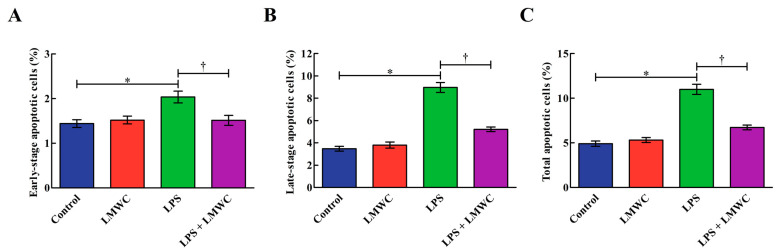
Effects of LMWC on apoptosis in LPS-treated IPEC-J2 cells. (**A**) Early-stage apoptotic cells. (**B**) Late-stage apoptotic cells. (**C**) Total apoptotic cells. Cells were pretreated with LMWC (400 μg/mL) for 2 h, followed by cotreatment with LPS (5 μg/mL) for another 6 h. Data are presented as the mean ± standard error (*n* = 4). * Means significant difference compared with the control group (*p* < 0.05). ^†^ Means significant difference compared with the LPS group (*p* < 0.05).

The percentages of early-stage, late-stage and total apoptotic cells in the LPS group were higher (*p* < 0.05) than those in the control group (Figure 5). In contrast, the percentages of early-stage, late-stage and total apoptotic cells in the LPS + LMWC group were lower (*p* < 0.05) than those in the LPS group.

### 2.6. Cleaved Cysteinyl Aspartate-Specific Protease (Caspase) Contents

**Figure 6:**
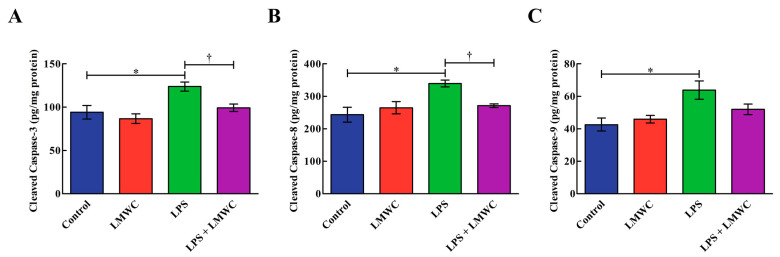
Effects of LMWC on the cleaved caspase contents in LPS-treated IPEC-J2 cells. (**A**) Cleaved caspase-3: cleaved cysteinyl aspartate-specific protease-3. (**B**) Cleaved caspase-8: cleaved cysteinyl aspartate-specific protease-8. (**C**) Cleaved caspase-9: cleaved cysteinyl aspartate-specific protease-9. Cells were pretreated with LMWC (400 μg/mL) for 2 h, followed by cotreatment with LPS (5 μg/mL) for another 6 h. Data are presented as the mean ± standard error (*n* = 4). * Means significant difference compared with the control group (*p* < 0.05). ^†^ Means significant difference compared with the LPS group (*p* < 0.05).

Following LPS stimulation, an increase (*p* < 0.05) in the cleaved caspase-3, -8 and -9 contents was observed in the IPEC-J2 cells (Figure 6). However, LMWC pretreatment reduced (*p* < 0.05) the cleaved caspase-3 and -8 contents in the LPS-treated IPEC-J2 cells.

### 2.7. Apoptosis-Related Gene Expression

**Figure 7:**
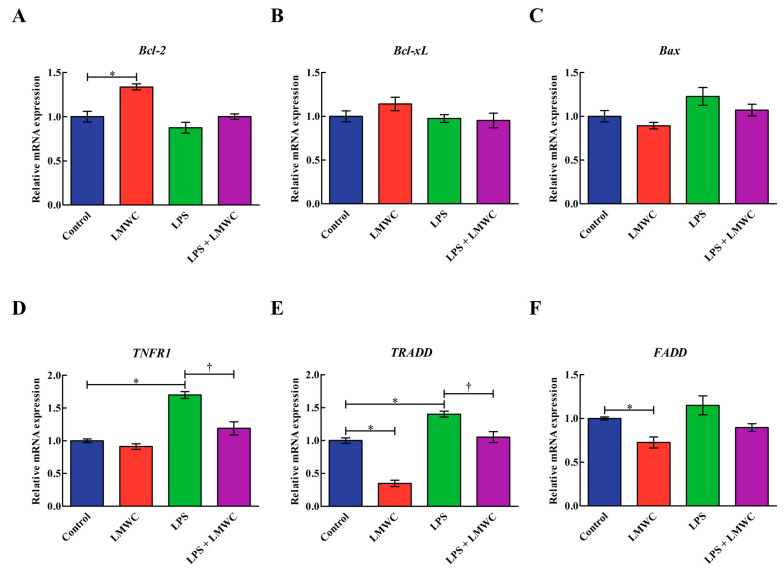
Effects of LMWC on the mRNA levels of apoptosis-related genes in LPS-treated IPEC-J2 cells. (**A**) *Bcl-2*: B-cell lymphoma-2. (**B**) *Bcl-xL*: Bcl-2-like-1. (**C**) *Bax*: B-cell lymphoma-2-associated X protein. (**D**) *TNFR1*: tumour necrosis factor receptor 1. (**E**) *TRADD*: tumour necrosis factor receptor-associated death domain. (**F**) *FADD*: Fas-associated death domain. Cells were pretreated with LMWC (400 μg/mL) for 2 h, followed by cotreatment with LPS (5 μg/mL) for another 6 h. Data are presented as the mean ± standard error (*n* = 4). * Means significant difference compared with the control group (*p* < 0.05). ^†^ Means significant difference compared with the LPS group (*p* < 0.05).

LMWC treatment upregulated (*p* < 0.05) the B-cell lymphoma-2 (*Bcl-2*) mRNA level but downregulated (*p* < 0.05) the TNF receptor-associated death domain (*TRADD*) and Fas-associated death domain (*FADD*) mRNA levels in the IPEC-J2 cells that were not treated with LPS (Figure 7). LPS treatment upregulated (*p* < 0.05) the TNF receptor 1 (*TNFR1*) and *TRADD* mRNA levels in the IPEC-J2 cells, while LMWC pretreatment downregulated (*p* < 0.05) the *TNFR1* and *TRADD* mRNA levels in the LPS-treated IPEC-J2 cells. The Bcl-2-like-1 (*Bcl-xL*) and Bcl-2-associated X protein (*Bax*) mRNA levels were unaffected (*p* > 0.05) by either LPS or LMWC.

### 2.8. Nuclear Factor-κB (NF-κB) Signalling Pathway-Related Protein Abundance 

**Figure 8:**
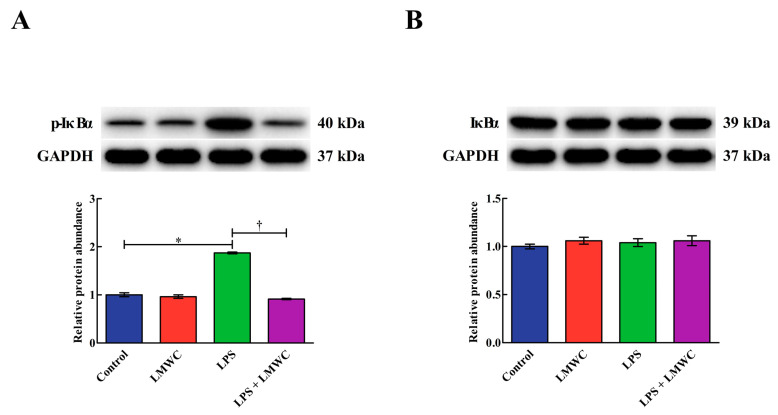
Effects of LMWC on the abundance of p-IκBα and IκBα protein in LPS-treated IPEC-J2 cells. (**A**) p-IκBα: phosphorylated inhibitor of nuclear factor-κB α. (**B**) IκBα: inhibitor of nuclear factor-κB α. Cells were pretreated with LMWC (400 μg/mL) for 2 h, followed by cotreatment with LPS (5 μg/mL) for another 6 h. Data are presented as the mean ± standard error (*n* = 4). * Means significant difference compared with the control group (*p* < 0.05). ^†^ Means significant difference compared with the LPS group (*p* < 0.05).

**Figure 9:**
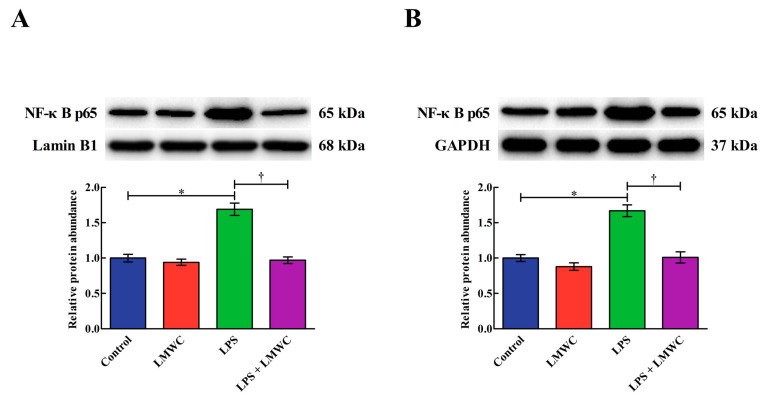
Effects of LMWC on the nuclear and cytoplasmic abundance of NF-κB p65 in LPS-treated IPEC-J2 cells. (**A**) Nuclear NF-κB p65: intra-nuclear nuclear factor-κB p65. (**B**) Cytoplasmic NF-κB p65: cytoplasmic nuclear factor-κB p65. Cells were pretreated with LMWC (400 μg/mL) for 2 h, followed by cotreatment with LPS (5 μg/mL) for another 6 h. Data are presented as the mean ± standard error (*n* = 4). * Means significant difference compared with the control group (*p* < 0.05). ^†^ Means significant difference compared with the LPS group (*p* < 0.05).

As shown in Figure 8, LPS stimulation increased (*p* < 0.05) the phosphorylated inhibitor of nuclear factor-κB α (p-IκBα) abundance in the IPEC-J2 cells, while LMWC pretreatment decreased (*p* < 0.05) the p-IκBα abundance in the LPS-treated IPEC-J2 cells. LMWC pretreatment decreased (*p* < 0.05) the nuclear and cytoplasmic abundance of NF-κB p65 in the LPS-treated IPEC-J2 cells (Figure 9).

## 3. Discussion

The intestinal epithelium forms a defence barrier to prevent antigens, pathogens and toxins in the intestinal lumen from entering the internal environment [19,20]. Tight junctions are an important part of the intestinal epithelial barrier; they are located in the apical membrane and basolateral surface of intestinal epithelial cells and are responsible for cell–cell adhesion, polarity and forming a permeability barrier for intercellular solute transport [21,22,23,24]. Of the tight junctions, occludin is involved in the regulation of intermembrane diffusion and paracellular diffusion of small molecules; it has functional importance in maintaining the integrity of the intestinal epithelial barrier [25]. In the present study, we found that LPS decreased the occludin abundance in IPEC-J2 cells, while LMWC pretreatment restored the LPS-induced reduction in the occludin abundance. As such, LMWC exerts beneficial effects in preventing LPS-induced tight junction injury in IPEC-J2 cells.

Previous studies have revealed that proinflammatory cytokines overproduction caused by LPS would induce intestinal epithelial cell damage [26,27]. In the present study, after LMWC pretreatment, the concentration of the proinflammatory cytokine TNF-α decreased in the LPS-stimulated IPEC-J2 cells, suggesting that LMWC can relieve LPS-induced inflammation in IPEC-J2 cells. Under normal circumstances, NF-κB is bound to IκBs (mainly IκBα) in the cytoplasm [28,29]. On stimulation with LPS, IκBs would phosphorylate and activated NF-κB would then enter the nucleus, thereby promoting the production of proinflammatory cytokines [30]. In the present study, we detected that LMWC not only decreased the p-IκBα abundance but also reduced the nuclear and cytoplasmic abundance of NF-κB p65 in the LPS-treated IPEC-J2 cells. These results indicate that LMWC decreases proinflammatory cytokine concentrations in LPS-stimulated IPEC-J2 cells via the NF-κB signalling pathway.

So far, two major signalling pathways are known to mediate apoptosis: intrinsic and extrinsic pathways [31]. The intrinsic pathway is initiated in the mitochondria and primarily leads to caspase-9 activation [32,33], while the extrinsic pathway is mediated by cell surface death receptors (e.g., TNFR1) and primarily leads to caspase-8 activation [34,35]. These apoptotic pathways converge on caspase-3 activation, which subsequently activates the remaining caspase cascade and results in apoptotic cell death [36]. In the present study, concurrent with the reduced apoptosis rate in the LMWC pretreated LPS-stimulated IPEC-J2 cells, we noted that LMWC pretreatment decreased the cleaved caspase-3 and -8 contents and *TNFR1* expression level in the LPS-treated IPEC-J2 cells. In summary, LMWC can reduce TNFR1-mediated apoptosis caused by LPS in IPEC-J2 cells, thereby ameliorating the LPS-induced inflammatory injury of IPEC-J2 cells.

In conclusion, LMWC could attenuate LPS-induced intestinal epithelial cell damage by preventing TNFR1-mediated apoptosis and reducing the secretion of proinflammatory cytokines via the NF-κB signalling pathway.

## 4. Material and Methods

### 4.1. Cell Culture

IPEC-J2 cells were kindly provided by the Institute of Subtropical Agriculture, Chinese Academy of Sciences (Changsha, China). The cells were grown in Dulbecco’s modified Eagle medium/Nutrient Mixture F-12 (DMEM/F12; Life Technologies Corporation, Grand Island, NY, USA) supplemented with 10% foetal bovine serum (Life Technologies Corporation) and 1% antibiotics (100 U/mL penicillin and 100 μg/mL streptomycin; Life Technologies Corporation) and maintained under a humidified atmosphere of 5% CO_2_ at 37 °C in a 25 cm^2^ cell culture flask (Corning Inc., Corning, NY, USA). At about 80–90% confluence, the IPEC-J2 cells were passaged by trypsinisation with 0.25% trypsin–EDTA.

### 4.2. LPS-Induced Inflammation Model Establishment

The IPEC-J2 cells were seeded in six-well cell culture plates (2 × 10^5^ cells/well), cultured overnight to allow cell attachment and incubated with different concentrations of LPS (0, 5, 10, 20 and 40 μg/mL, *Escherichia coli* O55:B5; Sigma-Aldrich, St. Louis, MO, USA) for specific durations (3, 6, 12 and 24 h). Finally, the IPEC-J2 cells were collected for gene expression analysis.

### 4.3. LMWC Dose Selection

The IPEC-J2 cells were seeded in six-well cell culture plates at a density of 2 × 10^5^ cells/well and then maintained at 37 °C in a humidified atmosphere of 5% CO_2_. After growing to subconfluence, the cells were pretreated with different concentrations of LMWC (0, 100, 200, 400, 600, 800 and 1000 μg/mL; Jiaxing Korui Biotech Co., Ltd., Jiaxing, China) for 2 h and then cotreated with or without LPS for another 6 h. Finally, the IPEC-J2 cells were collected for gene expression analysis.

### 4.4. Drug Treatment

The IPEC-J2 cells were seeded in six-well culture plates at a density of 2 × 10^5^ cells/well until they reached 80–90% confluence. The cells were pretreated with LMWC for 2 h. Subsequently, in the presence or absence of LMWC, the IPEC-J2 cells were treated with or without LPS for 6 h. After the treatment, the IPEC-J2 cells and cell cultures were collected for further analysis.

### 4.5. RNA Extraction, cDNA Synthesis and Quantitative Real-Time PCR (qPCR)

After the IPEC-J2 cells were washed twice with ice-cold PBS, RNAiso Plus (Takara Biotechnology Co., Ltd., Dalian, China) was used to extract the total RNA according to the manufacturer’s instructions. The RNA concentration and purity were determined using a NanoDrop 2000 spectrophotometer (Thermo Fisher Scientific, Inc., Waltham, MA, USA). Reverse transcription was performed with 1 μg of total RNA using the PrimeScript™ RT Reagent Kit with gDNA Eraser (Takara Biotechnology Co., Ltd.) to generate cDNA.

All qPCR reactions were performed in duplicate on the CFX96 Real-Time PCR System (Bio-Rad Laboratories, Inc., Hercules, CA, USA) using TB Green™ Premix Ex Taq™ II (Tli RNaseH Plus; Takara Biotechnology Co., Ltd.). For each PCR reaction, 1 μL cDNA, 5 μL TB Green Premix Ex Taq II (Tli RNaseH Plus, 2×), 0.5 μL each of forward primer and reverse primer (the primer sequences are listed in Table 1) and 3 μL diethylpyrocarbonate-treated water were directly added to the PCR reaction mixture and set to a final volume of 10 μL. The reaction conditions were as follows: 1 cycle at 95 °C for 30 s, followed by 40 cycles at 95 °C for 5 s and 60 °C for 34 s. Following each qPCR reaction, a melting curve analysis was performed at 60–95 °C with an increment of 0.5 °C/5 s in order to check the specificity of the PCR products. For determining the efficiency of the PCR reactions, standard curves were obtained for each gene using 10-fold serial dilutions (six points). After verifying that the primers were amplified with amplification efficiency values of about 100%, the 2^−ΔΔCT^ method was used to analyse the relative mRNA levels of all the target genes [37].

### 4.6. Cytokine Concentration Analysis

The cell cultures were centrifuged at 3000× *g* at 4 °C for 15 min to acquire culture supernatants. Before the IL-1, IL-6, TNF-α and IFN-γ concentrations in the culture supernatants were measured using ELISA kits (Jiangsu Jingmei Biotechnology Co., Ltd., Yancheng, China), the protein concentration in the culture supernatants was assessed. Moreover, the cytokine concentrations were normalised to the corresponding protein concentrations.

### 4.7. Cell Apoptosis Detection

Apoptotic cells were detected using the PE Annexin V Apoptosis Detection Kit I (Becton, Dickinson and Company, BD Biosciences, San Jose, CA, USA) according to the manufacturer’s instructions. In brief, the IPEC-J2 cells were harvested with 0.25% trypsin without EDTA, collected in centrifuge tubes and centrifuged at 350× *g* at 4 °C for 10 min. The IPEC-J2 cells were washed twice in ice-cold PBS, and the supernatants were removed. Following this, 5 μL Annexin V-FITC and 5 μL 7-aminoactinomycin D were added to the cell suspension and incubated for 15 min at room temperature in the dark. Finally, 400 μL annexin V binding buffer (1×) was added to the mixture and cell apoptosis was evaluated using a CytoFlex flow cytometer (Beckman Coulter, Inc., Brea, CA, USA).

### 4.8. Cleaved Caspase Content Measurement

The IPEC-J2 cells were harvested with 0.25% trypsin–EDTA, collected in centrifuge tubes and centrifuged at 600× *g* at 4 °C for 5 min. The supernatants were removed, following which the IPEC-J2 cells were washed once with ice-cold PBS, lysed in 100 μL lysis buffer on ice for 30 min and the supernatants of the centrifuge (16,000× *g* at 4 °C for 15 min) were obtained. Next, the cleaved caspase-3, -8 and -9 contents in the supernatants of the centrifuge were determined using ELISA kits (Jiangsu Jingmei Biotechnology Co., Ltd.) according to the manufacturer’s instructions.

### 4.9. Western Blot Analysis

Total proteins from the IPEC-J2 cells were extracted using RIPA buffer containing phenylmethylsulfonyl fluoride (Beyotime Institute of Biotechnology, Shanghai, China). Nuclear and cytoplasmic protein fractions were extracted from the IPEC-J2 cells using NE-PER^TM^ nuclear and cytoplasmic extraction reagents (Thermo Fisher Scientific, Inc.), respectively. The samples were transferred to 1.5 mL centrifuge tubes and centrifuged at 13,000× *g*/4 °C for 15 min to obtain supernatants. The supernatants were collected for determining the protein concentration using the bicinchoninic acid method [38] and then diluted with 4× Laemmli sample buffer (Bio-Rad Laboratories, Inc.) containing 10% β-mercaptoethanol and denatured at 95 °C for 10 min. Equal amounts of protein lysates were loaded, separated by sodium dodecyl sulphate-polyacrylamide gel electrophoresis and transferred onto polyvinylidene fluoride (PVDF) membranes (Merck Millipore Ltd., Tullagreen, Ireland). The PVDF membranes were blocked with 5% non-fat dry milk at room temperature for 1 h, and then incubated at 4 °C overnight with specific primary antibodies, including rabbit anti-occludin antibody (Abcam plc., Cambridge, UK), rabbit anti-p-IκBα antibody (Thermo Fisher Scientific, Inc.), mouse anti-IκBα antibody (Cell Signalling Technology, Inc., Danvers, MA, USA), mouse anti-NF-κB p65 antibody (Cell Signalling Technology, Inc.), rabbit anti-lamin B1 antibody (Abcam plc.) and rabbit anti-GAPDH antibody (Cell Signalling Technology, Inc.). After being washed thrice with TBS/T, the PVDF membranes were incubated with the corresponding secondary antibodies, namely horseradish peroxidase-linked goat anti-rabbit IgG antibody (Cell Signalling Technology, Inc.) or horseradish peroxidase-linked horse anti-mouse IgG antibody (Cell Signalling Technology, Inc.), at room temperature for 1 h. Following this, the PVDF membranes were washed thrice with TBS/T and the protein bands were visualised using the Clarity™ Western ECL Substrate (Bio-Rad Laboratories, Inc.) via the ChemiDoc^TM^ XRS+ Imager System (Bio-Rad Laboratories, Inc.). The protein bands were analysed using Quantity One software (version 3.0; Bio-Rad Laboratories, Inc.); the results are expressed as the ratio of targeted protein to reference protein.

### 4.10. Statistical Analysis

All the data are presented as the mean ± standard error. The comparison of two means was performed using Student’s *t*-test of SAS software (version 9.0; SAS Institute, Inc., Cary, NC, USA), while the comparison of more than two groups was performed by a one-way analysis of variance using the general linear model procedure of SAS software (version 9.0; SAS Institute, Inc.). Differences were considered statistically significant at *p* < 0.05.

## Figures and Tables

**Table 1:** Primer sequences for qPCR.

Gene *	Primer Sequence (5′–3′)	Size (bp)	Accession No.
*IL-1β*	Forward: GAAAGATAACACGCCCACCC Reverse: TCTGCTTGAGAGGTGCTGATGT	165	NM_214055.1
*IL-6*	Forward: CCTGTCCACTGGGCACATAAC Reverse: CAAGAAACACCTGGCTCTGAAAC	252	NM_214399.1
*TNF-α*	Forward: CATCGCCGTCTCCTACCA Reverse: CCCAGATTCAGCAAAGTCCA	199	NM_214022.1
*IFN-γ*	Forward: GAGCCAAATTGTCTCCTTCTAC Reverse: CGAAGTCATTCAGTTTCCCAG	140	NM_213948.1
*Bcl-2*	Forward: AGCATGCGGCCTCTATTTGA Reverse: GGCCCGTGGACTTCACTTAT	120	XM_021099593.1
*Bcl-xL*	Forward: GGTCGCATTGTGGCCTTTTT Reverse: CGTCAGGAACCATCGGTTGA	237	NM_214285.1
*Bax*	Forward: CTGACGGCAACTTCAACTGG Reverse: CGTCCCAAAGTAGGAGAGGA	200	XM_003127290.5
*TNFR1*	Forward: CTGGCATTCTTCCTCTTCGTTG Reverse: CCGGCTCTCCCTCCTTTACA	109	NM_213969.1
*TRADD*	Forward: AGGCGTGCTTGGAGGCT Reverse: GCGAAGATGAAATTCAAACAGC	124	XM_021094047.1
*FADD*	Forward: CTGCGACAACGTGGGGA Reverse: TCAGGTTTCGGGGATACTTC	101	NM_001031797.1
*GAPDH*	Forward: ATGGTGAAGGTCGGAGTGAAC Reverse: CTCGCTCCTGGAAGATGGT	235	NM_001206359.1

*IL-1β: interleukin-1β. *IL-6*: interleukin-6. *TNF-α*: tumour necrosis factor-α. *IFN-γ*: interferon-γ. *Bcl-2*: B-cell lymphoma-2. *Bcl-xL*: Bcl-2-like-1. *Bax*: B-cell lymphoma-2-associated X protein. *TNFR1*: tumour necrosis factor receptor 1; *TRADD*: tumour necrosis factor receptor-associated death domain. *FADD*: Fas-associated death domain. *GAPDH*: glyceraldehyde-3-phosphate dehydrogenase.

## Data Availability

The data presented in this study are available on request from the corresponding author.

## Sample Availability

Samples of the compound that deacetylated from chitosan (LMWC) are available from the authors.
